# Etymologia: *Cronobacter sakazakii*

**DOI:** 10.3201/eid2411.ET2411

**Published:** 2018-11

**Authors:** Ronnie Henry

**Keywords:** Cronobacter sakazakii, bacteria, Enterobacter, Riichi Sakazaki, John J. Farmer, Cronos, dried milk, powdered formula, neonatal meningitis, sepsis

## *Cronobacter sakazakii* [kroʹno-bakʺtər sakʹǝ-zakʺee-ī]

The first documented isolation of what would become known as *Cronobacter sakazakii* was from a can of dried milk in 1950, although these organisms have likely existed for millions of years. In 1980, John J. Farmer III, proposed the name *Enterobacter sakazakii* for what had been known as “yellow-pigmented *E. cloacae*,” in honor of Japanese bacteriologist Riichi Sakazaki. Over the next decades, *E. sakazakii* was implicated in scores of cases of meningitis and sepsis among infants, frequently in association with powdered infant formula. In 2007, the genus *Cronobacter* was created to accommodate the biogroups of *E. sakazakii*, with *C. sakazakii* as the type species. The genus was named for Cronos, the Titan of Greek myth, who devoured his children as they were born (Figure).

**Figure:**
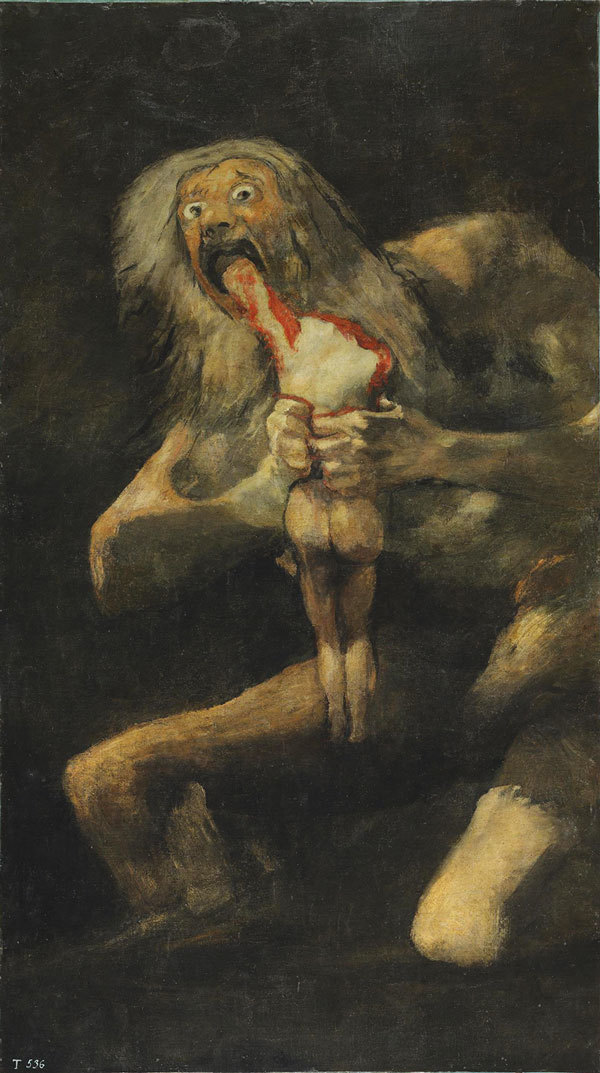
Francisco Goya (1746–1828), Saturn Devouring His Son, 1819–1823, oil mural transferred to canvas, via Wikimedia Commons.
